# Transcriptome Analysis Reveals the Profile of Long Non-Coding RNAs during Myogenic Differentiation in Goats

**DOI:** 10.3390/ijms24076370

**Published:** 2023-03-28

**Authors:** Chenyu Yang, Xinyi Zhou, Yanan Xue, Dandan Li, Linjie Wang, Tao Zhong, Dinghui Dai, Jiaxue Cao, Jiazhong Guo, Li Li, Hongping Zhang, Siyuan Zhan

**Affiliations:** 1 Farm Animal Genetic Resources Exploration and Innovation Key Laboratory of Sichuan Province, College of Animal Science and Technology, Sichuan Agricultural University, Chengdu 611130, China; 2 Key Laboratory of Livestock and Poultry Multi-Omics, Ministry of Agriculture and Rural Affairs, College of Animal Science and Technology, Sichuan Agricultural University, Chengdu 611130, China

**Keywords:** lncRNAs, transcriptome, differential expression, myogenic differentiation, goats

## Abstract

The long non-coding RNAs (lncRNAs) are emerging as essential regulators of the growth and development of skeletal muscles. However, little is known about the expression profiles of lncRNAs during the proliferation and differentiation of skeletal muscle satellite cells (MuSCs) in goats. In this study, we investigate potential regulatory lncRNAs that govern muscle development by performing lncRNA expression profiling analysis during the proliferation (cultured in the growth medium, GM) and differentiation (cultured in the differentiation medium, DM1/DM5) of MuSCs. In total, 1001 lncRNAs were identified in MuSC samples, and 314 differentially expressed (DE) (FDR < 0.05, |log2FC| > 1) lncRNAs were screened by pairwise comparisons from three comparison groups (GM-vs-DM1, GM-vs-DM5, DM1-vs-DM5). Moreover, we identified the cis-, trans-, and antisense-regulatory target genes of DE lncRNAs. Gene Ontology (GO) and Kyoto Encyclopedia of Genes and Genomes (KEGG) enrichment analyses showed that these target genes were significantly enriched in muscle development-related GO terms and KEGG pathways. In addition, the network of interactions between DE lncRNAs and their target genes was identified, which included well-known myogenesis regulators such as Myogenic differentiation 1 (MyoD), Myogenin (MyoG), and Myosin heavy chain (MyHC). Meanwhile, competing endogenous RNA (ceRNA) network analysis showed that 237 DE lncRNAs could bind to 329 microRNAs (miRNAs), while miRNAs could target 564 mRNAs. Together, our results provide a genome-wide resource of lncRNAs that may contribute to myogenic differentiation in goats and lay the groundwork for future investigation into their functions during skeletal muscle development.

## 1. Introduction

The quantity and quality of the skeletal muscle of livestock play a very important part in determining the quality of the meat [1]. Therefore, the study of skeletal muscle growth and development is very important for breeding in the field of animal husbandry. However, skeletal muscle growth and development is a complex process that involves the activation of skeletal muscle satellite cells (MuSCs), the proliferation and differentiation of myoblasts, and the fusion of myoblasts into myofibers, as well as the expression of a wide range of genes [2,3]. To make the research of molecular breeding more in-depth, it is necessary to push the research perspective to the cellular and molecular level, which makes the study of the proliferation and differentiation of MuSCs become a hot spot. Additionally, it is important to note that the high species specificity of primary cultured myoblasts makes them an ideal model for studying the proliferation and differentiation of muscle cells in vitro, allowing us to more easily approach the mystery and landscape of skeletal muscle development.

The long non-coding RNAs (lncRNAs) are a class of non-coding RNAs (ncRNAs) with a length greater than 200 nucleotides. In recent years, increasing evidence indicates that lncRNAs play an essential role in the growth and development of skeletal muscle cells in animals, thereby regulating muscle activity [4,5,6,7,8]. For example, lncRNA lnc-mg plays a key role in promoting myogenesis by functioning as a competing endogenous RNA (ceRNA) for microRNA-125b, thus controlling the amount of insulin-like growth factor 2 (IGF-2) protein [9]. LncMyoD can be activated directly by Myogenic differentiation 1 (MyoD) and then it binds to IGF2-mRNA-binding protein 2 (IMP2), thus limiting the translation of proliferation genes like N-Ras and c-Myc during myoblast development [10]. SYNPO2 intron sense overlapping lncRNA (lnc-SYISL) is capable of interacting directly with the enhancer of the polycomb repressive complex 2 (PRC2), regulating p21 expression and muscle-specific genes, promoting myoblast differentiation, and inhibiting myoblast proliferation [11]. Additionally, there are many modes of action for lncRNAs on gene expression during skeletal myogenesis, which can be cis-acting, trans-acting, antisense-acting, and so on. For instance, lncRNA Six1 is associated with the Six1 gene and encodes a micro-peptide that is used to activate Six1 in cis, thereby increasing muscle cell proliferation and participating in muscle development [12]. In addition, it has been reported that lncRNA H19 has the potential to be used as an IGF2 trans-regulator during muscle development [13]. On the other hand, a set of antisense lncRNAs related to some important pathways involved in fetal myogenesis and muscle cell differentiation were identified by using a bioinformatics approach in our previous work [14]. In a word, it is possible to gain more insights into how lncRNA regulates the proliferation and differentiation of MuSCs, as well as the development of muscle by analyzing both cis-, trans-, and antisense-acting interactions between lncRNA and mRNA.

There is no doubt that the differentiation of myoblasts and their fusion into multinucleate myotubes is a crucial step in the development of skeletal muscle. However, most published studies on the lncRNAs of domestic animals are conducted by using skeletal muscle tissue. To gain a deeper understanding of how gene expression is controlled during myoblast differentiation into myotubes in goat muscle, we conducted a comprehensive analysis of lncRNAs and their interaction networks with mRNAs during myogenic differentiation of goat MuSCs. Our findings may provide abundant information about the modulation of muscle differentiation by lncRNAs and provide new outlooks for the development of skeletal muscles.

## 2. Results

### 2.1. Establishment of Goat MuSCs Differentiation Model

**Figure 1:**
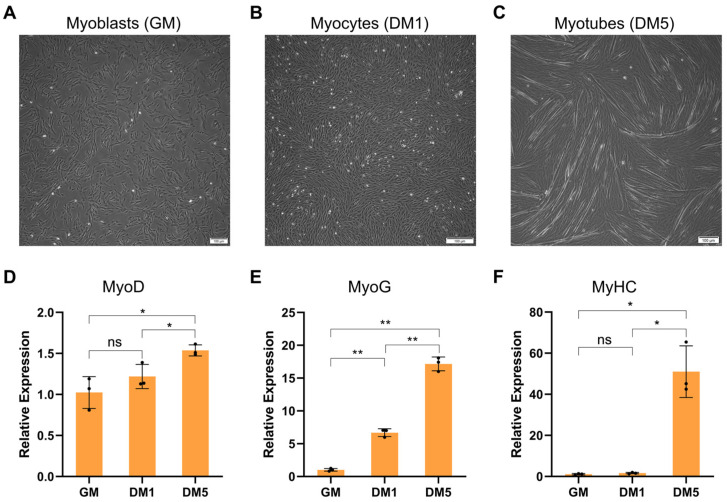
Morphological and gene expression characteristics of proliferating and differentiating goat MuSCs in vitro. (**A**) MuSCs were cultured in the growth medium until they achieved 80% confluence (GM, myoblasts). (**B**) MuSCs were cultured in the differentiation medium for 1 day (DM1, myocytes). (**C**) MuSCs were cultured in the differentiation medium for 5 days (DM5, myotubes). Scale bars = 100 µm. (**D**–**F**) Expression profiles of MyoD, MyoG, and MyHC from proliferation (GM) to differentiation (DM1 and DM5) in MuSCs. Data are the means ± SEM. * *p* < 0.05, ** *p* < 0.01.

The proliferation phase was defined as the MuSCs cultured in the growth medium (GM) with a confluence rate of around 80%. It was observed that myoblasts displayed a typical morphology with a unique shuttle shape, as well as a high proliferating ability (Figure 1A). Further, when the GM was replaced with the differentiation medium (DM), the cells proceeded to undergo multiple rounds of cell division and eventually differentiated into mononuclear myocytes. Upon one day, the myocytes started showing myotube fusion (DM1 samples, Figure 1B). On the fifth day, the myocytes united to form multinucleated myotubes, and the number of myotubes increased (DM5 samples, Figure 1C). To further identify gene expression characteristics of MuSCs, the expression levels of MyoD (an activation marker of MuSCs), MyoG (Myogenin, a marker of myogenic differentiation), and MyHC (Myosin heavy chain, a marker of myogenic differentiation) during MuSC differentiation were quantified by quantitative real-time PCR (qRT-PCR) (Figure 1D–F). The expression pattern of these marker genes during MuSC differentiation agreed with a previous study [15,16] and demonstrated that the MuSC differentiation model was successfully constructed.

### 2.2. Overview of RNA Sequencing Data

In total, 721,950,292 raw reads were generated in the nine libraries, and 720,121,930 clean reads were obtained after removing adaptor sequences and low-quality reads (Supplementary Table S1). As a result, the percentage of clean reads in all libraries ranged from 99.72 to 99.79%, and approximately 89.61 to 91.04% were uniquely mapped to the *Capra hircus* reference genome across all libraries (assembly ARS1) (Supplementary Table S1). The mean GC content of the nine libraries was 50.29%, and the Q30 of each sample was not less than 93.74% (Supplementary Table S1), indicating that the sequencing data were highly reliable and could be used for further analysis.

### 2.3. Identification and Characterization of LncRNAs in Goat MuSCs

**Figure 2:**
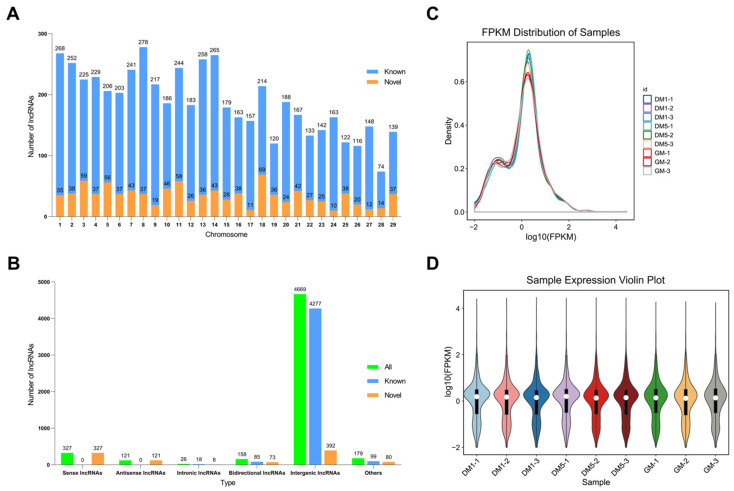
Identification and characterization of lncRNA in goat MuSCs. (**A**) Type statistics of identified lncRNAs. (**B**) Chromosome distribution of identified lncRNAs. (**C**) The density distribution of lncRNAs was according to log10(FPKM). (**D**) The nine sample expressions in a violin plot, which was replaced by log10(FPKM).

After analyzing the coding ability of 10,163 new transcripts by using Coding-Non-Coding-Index (CNCI) (v2) and Coding Potential Calculator (CPC) (v0.9-r2), RNA-seq yielded 1001 novel lncRNA transcripts (Supplementary Table S2). The 1001 novel lncRNA transcripts included 392 intergenic lncRNAs (39.16%), 327 sense lncRNAs (32.67%), 121 antisense lncRNAs (12.09%), 73 bidirectional lncRNAs (7.29%) and 8 intronic lncRNAs (0.80%) (Figure 2A). It is worth mentioning that all the sense lncRNAs and antisense lncRNAs are novel. LncRNA transcripts were randomly distributed on 29 autosomes (Figure 2B). FPKM was used to estimate the expression levels of lncRNA transcripts (Supplementary Table S3). Afterward, we evaluated the differences between the samples using a density distribution plot of the lncRNA and a violin plot of the expression of the nine samples. As a result, the similarity of expression density and expression level across nine samples indicates their similarity in library construction, sequencing, alignment, and quantification, further supporting the reliability of RNA-seq data (Figure 2C,D).

### 2.4. Analysis of Differential Expression of LncRNAs

**Figure 3:**
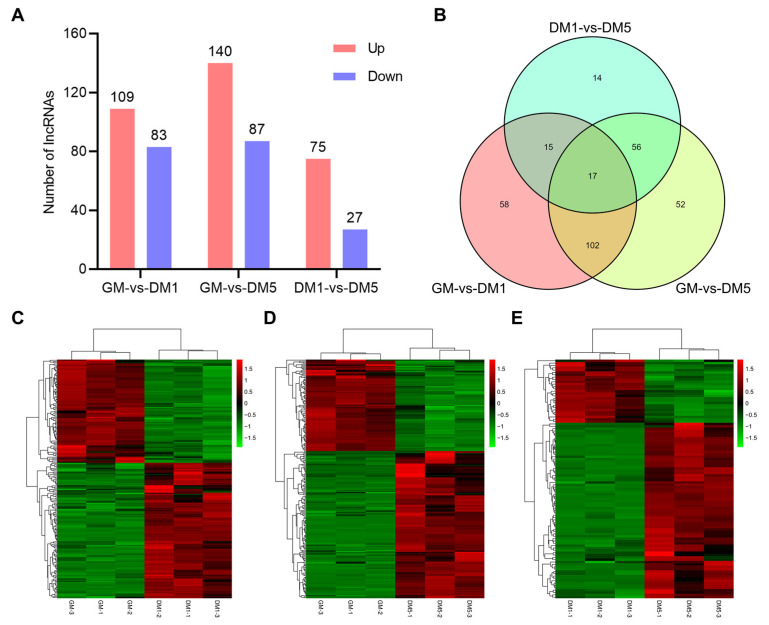
Analysis of DE lncRNAs. (**A**) Numbers of up-regulated and down-regulated lncRNAs. (**B**) Venn diagram showing the DE lncRNAs at the three comparisons. (**C**–**E**) Hierarchical clustering analysis of DE lncRNAs through pairwise comparisons. Red: relatively high expression; Green: relatively low expression.

During myogenic differentiation, 314 lncRNA transcripts were differentially expressed based on the differential analysis (FDR < 0.05, |log2FC| > 1) (Supplementary Table S4). Across all comparisons, the number of up-regulated differentially expressed (DE) lncRNAs was greater than the number of down-regulated DE lncRNAs (Figure 3A). The DE lncRNAs were further analyzed by constructing a Venn diagram using 192, 227, and 102 DE lncRNAs in GM-vs-DM1, GM-vs-DM5, and DM1-vs-DM5, respectively. A total of 58, 52, and 14 group-specific DE lncRNAs were detected in GM-vs-DM1, GM-vs-DM5, and DM1-vs-DM5, respectively, and 17 DE lncRNAs were detected in all three comparisons (Figure 3B and Supplementary Table S5). Finally, hierarchical clustering analysis was used to assess the expression patterns of DE lncRNAs to explore the similarities and to compare the relationships between the different libraries, and it was found that samples within the same group were well clustered together (Figure 3C–E).

### 2.5. Enrichment Analysis of Cis-Target Genes of LncRNAs

**Figure 4:**
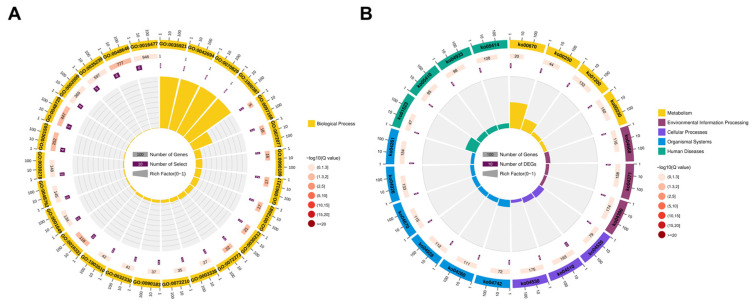
Enrichment analysis of cis-target genes of DE lncRNAs. (**A**) The top 26 GO terms of cis-target genes. (**B**) The top 20 KEGG pathways of cis-target genes. There are four circles from outside to inside. The first circle is the classification of GO or KEGG enrichment terms. Different colors represent different categories. The second circle shows the total number of foreground genes. The third circle represents the Q value and the number of background genes in the category. The fourth circle is the Rich Factor value of each category (the number of foreground genes in the category divided by the number of background genes). Each grid of the background auxiliary line represents 0.1.

According to our study, we found 984 lncRNAs that were transcribed close to 969 protein-coding neighbors (1285 pairs), including 36 DE lncRNAs that were transcribed close to 33 DE protein-coding neighbors (39 pairs) (Supplementary Table S6). Next, Gene Ontology (GO) analysis of the cis-target genes of DE lncRNAs was performed to explore their possible functions. The results showed that the cis-target genes were enriched in 358 GO terms related to a wide range of biological processes after filtering using the significant criteria of corrected *p* < 0.05 (Supplementary Table S7). As shown in Figure 4A, the top 26 GO terms are listed. In this list, three GO terms related to muscle development were found, including mesenchymal cell differentiation (GO:0048762), cell migration (GO:0016477), and muscle cell fate specification (GO:0042694). Furthermore, Kyoto Encyclopedia of Genes and Genomes (KEGG) pathway analysis was performed on the cis-target genes to identify pathways that were enriched. Several of the cis-target genes were enriched in 30 signaling pathways, including those related to muscle development, such as the focal adhesion, MAPK signaling pathway, Rap1 signaling pathway, and PI3K-Akt signaling pathway (Supplementary Table S7). The top 20 KEGG pathways associated with DE lncRNA cis-target genes are shown in Figure 4B. The results suggest that DE lncRNAs may be involved in the development of muscle cells.

### 2.6. Enrichment Analysis of Trans-Target Genes of LncRNAs

**Figure 5:**
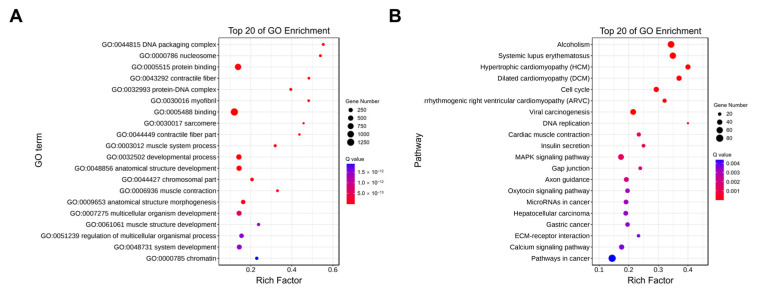
Enrichment analysis of trans-target genes of DE lncRNAs. (**A**) The top 20 significant GO terms. (**B**) The top 20 significant KEGG pathways. The color of the circle represents the Q value. The size of the circle indicates the number of target genes.

As a result of the trans-regulatory relationships predicted, 34,844 interaction relationships (26,034 positive and 8810 negative correlations) were found between 289 DE lncRNAs and 1932 DE mRNAs (Supplementary Table S8). According to functional analysis, the co-expressed genes are significantly enriched in 1949 GO terms (1489 under biological process, 209 under cellular component, and 251 under molecular function) (*p* < 0.05) (Supplementary Table S9). As shown in Figure 5A, the top 20 GO terms for trans-target genes of DE lncRNAs are outlined. It is noteworthy that some of these terms relate to the development of skeletal muscle, such as myofibril (GO:0030016), sarcomere (GO:0030017), muscle system process (GO:0003012), muscle contraction (GO:0006936), striated muscle tissue development (GO:0014706), muscle cell development (GO:0055001), and muscle cell differentiation (GO:0042692). In addition, the co-expressed genes were significantly enriched in 79 KEGG pathways (*p* < 0.05), several of which were related to muscle cell development, such as the cell cycle, MAPK signaling pathway, gap junctions, and calcium signaling pathways (Supplementary Table S9). The top 20 KEGG pathways for the trans-target genes of DE lncRNAs are shown in Figure 5B. These findings indicate that lncRNAs are also involved in muscle development by regulating trans-target genes.

### 2.7. Enrichment Analysis of Antisense-Target Genes of LncRNAs

According to antisense lncRNA analysis, 125 interactions were detected between 121 lncRNAs and 112 mRNAs, and 9 interactions were detected between DE lncRNAs and DE mRNAs (Supplementary Table S10). By analyzing the antisense-target genes of DE lncRNAs, 249 GO terms (183 under biological process, 5 under cellular component, and 61 under molecular function) and 18 KEGG pathways were significantly enriched (*p* < 0.05) (Supplementary Table S11 and Supplementary Figure S1A,B). In this list, there are several GO terms related to muscle development, including cardiac muscle cell proliferation (GO:0060038), cardiac muscle tissue growth (GO:0055017), and striated muscle cell proliferation (GO:0014855). In addition, ABC transporter and TGF-beta signaling pathways were also identified as KEGG pathways related to muscle cell development.

### 2.8. Construction of DE LncRNA-mRNA Interaction Networks

**Figure 6:**
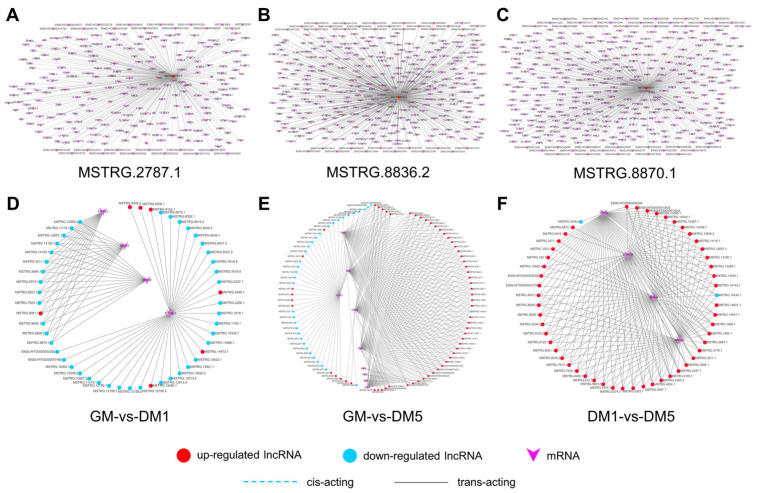
The lncRNA-mRNA interaction networks. (**A**–**C**) LncRNA-mRNA networks for MSTRG.2787.1, MSTRG.8836.2, and MSTRG.8870.1. (**D**–**F**) LncRNA-mRNA networks associated with MyoD, MyoG, and MyHC in GM-vs-DM1, GM-vs-DM5, and DM1-vs-DM5. DE lncRNAs and their corresponding target genes were used to establish the lncRNA-gene interaction networks. In these networks, genes are displayed in purple arrow, while lncRNAs are displayed in circle; up-regulated lncRNA is indicated with red, while down-regulated lncRNA is indicated with blue. The cis-acting interactions are represented as dashed lines, whereas the trans-acting interactions are represented as solid lines.

The network of DE lncRNA-mRNA interaction was established using DE lncRNAs and the corresponding target genes to evaluate how lncRNAs regulate muscle differentiation. The network analysis focused on three DE lncRNAs (MSTRG.2787.1, MSTRG.8836.2, and MSTRG.8870.1) that interacted with more target genes, which probably constitute the center of the network (Figure 6A–C). The number of target genes for MSTRG.2787.1, MSTRG.8836.2, and MSTRG.8870.1 is 239, 335, and 359, respectively. Additionally, these three lncRNAs were up-regulated during the myogenic differentiation of goat MuSCs, which suggests that they may be involved in myogenic differentiation.

Furthermore, we focus on DE lncRNAs associated with MyoD, MyoG, and MyHC in three comparison groups (Figure 6D–F). In GM-vs-DM1, we identified 17,177 interaction pairs (21 cis-acting pairs and 17,156 trans-acting pairs) between 180 DE lncRNAs and 1177 corresponding target genes, including 14 MyoG-related pairs, 36 MyoD-related pairs, and 28 MyHC-related pairs (Supplementary Table S12). A total of 28,310 interaction pairs (25 cis-acting pairs and 28,285 trans-acting pairs) were found between 219 DE lncRNAs and their corresponding 1338 targets in GM-vs-DM5, of which 44 are MyoG-related, 42 are MyoD-related, and 194 are MyHC-related pairs (Supplementary Table S12). In DM1-vs-DM5, we identified 13,822 interactions (13 cis-acting pairs and 13,809 trans-acting pairs) between 94 DE lncRNAs and 730 target genes, including 44 MyoG-related pairs, 146 MyHC-related pairs, and no MyoD-related pairs (Supplementary Table S12). As a result of the analysis, we identified seven lncRNAs (MSTRG.3694.1, MSTRG.8870.1, MSTRG.5374.1, MSTRG.10559.2, MSTRG.5087.1, MSTRG.12657.1, MSTRG.4037.1) that were shown to have more interactions, suggesting that they may play a significant role in the differentiation of myoblasts.

### 2.9. Construction of Potential LncRNA-miRNA-mRNA Regulatory Networks

**Figure 7:**
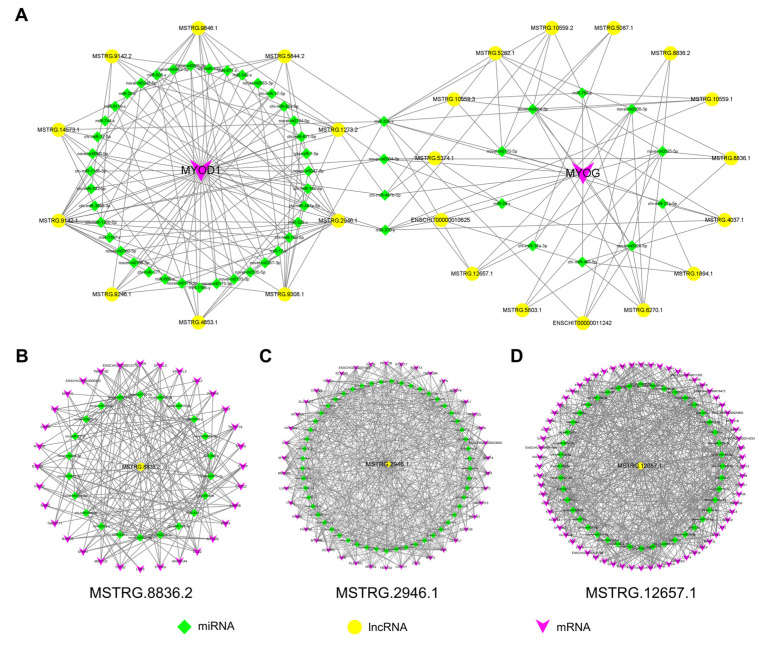
The ceRNA networks. (**A**) CeRNA networks associated with MyoD and MyoG. (**B**–**D**) CeRNA networks for MSTRG.8836.2, MSTRG.2946.1, and MSTRG.12657.1. DE lncRNAs and mRNAs, as well as their common target miRNAs were used to establish the ceRNA networks. In these networks, genes are displayed in purple arrow, lncRNAs are displayed in yellow circle, and miRNAs are indicated with green diamond patterns.

The ceRNA network of lncRNA-miRNA-mRNA was constructed to determine the key lncRNAs related to goat skeletal myogenesis. The resulted ceRNA networks included 237 DE lncRNAs, 329 microRNAs (miRNAs), and 564 mRNAs (Supplementary Table S13). First, we focus on the ceRNA network associated with MyoD and MyoG, which includes 25 lncRNAs and 53 miRNAs (Figure 7A). It is noteworthy that MSTRG.8836.2 was identified again, suggesting that it plays a critical role in myogenesis. Additionally, ceRNAs that showed high connectivity (the number of co-expressed targeted miRNAs) were regarded as hub genes that play an important role in biological networks (Supplementary Table S14). A total of 5 of the top 10 lncRNAs (MSTRG.2946.1, MSTRG.9142.1, MSTRG.5262.1, MSTRG.12657.1, MSTRG.4037.1), which were up-regulated in all three comparison groups, target the same miRNAs as either MyoD or MyoG. Furthermore, three regulatory networks based on the connectivity analysis were constructed for MSTRG.8836.2, MSTRG.2946.1, and MSTRG.12657.1, which represent potential regulators of goat myogenic differentiation (Figure 7B–D).

### 2.10. Validation of LncRNAs by qRT-PCR

**Figure 8:**
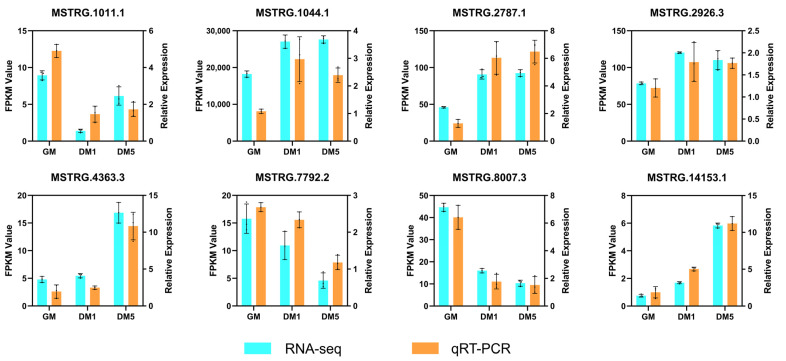
Validation of eight lncRNAs by qRT-PCR. Expression levels were measured by RT-qPCR, calculated by the 2-ΔΔCt, and normalized to GAPDH. Data are the mean ± SEM.

To verify the reliability of RNA-seq data, eight lncRNAs (MSTRG.1011.1, MSTRG.1044.1, MSTRG.2787.1, MSTRG.2926.3, MSTRG.4363.3, MSTRG.7792.2, MSTRG.8007.3, and MSTRG.14153.1) were randomly selected for measuring expression levels by qRT-PCR. The results showed that expression patterns of lncRNAs in qRT-PCR were consistent with those found in RNA-seq, further demonstrating the accuracy and reliability of the sequencing data (Figure 8).

## 3. Discussion

Skeletal muscle development requires the precise regulation of protein-coding genes, ncRNAs, and numerous other factors. As one of the most important members of the ncRNA family, lncRNA plays a vital role in regulating the transcriptional and post-transcriptional expression of genes in muscles [7,17]. In the past, numerous lncRNAs have been discovered in livestock, and these molecules are demonstrated to have a key role in the development of a variety of tissues [6,8,18,19,20]. However, there are few studies on lncRNA transcriptome in muscle cell development, since most studies are conducted at the tissue level. In this study, we determined the expression profiles of lncRNAs in goat MuSCs during myogenic differentiation. During this process, a total of 1001 lncRNAs were identified, and 314 lncRNAs were differentially expressed. Importantly, 17 lncRNAs were found to be differentially expressed across all three comparisons, suggesting their critical roles in muscle differentiation.

The functions of lncRNAs, different from miRNAs and proteins, cannot be inferred from their sequence or structure. Therefore, we predicted the potential functions of lncRNAs using cis, trans, and antisense methods. Enrichment analysis of the cis-target genes revealed that DE lncRNAs were enriched in muscle development-related GO terms and KEGG pathways, such as muscle cell fate specification, skeletal muscle cell differentiation, MAPK signaling pathway, and PI3K-Akt signaling pathway. The results suggest that DE lncRNAs may be involved in the development of muscle cells through cis-regulation. Indeed, it has been demonstrated that lncRNA Dum promotes myoblast differentiation and damage-induced muscle regeneration by silencing its neighboring gene, Dppa2, in cis by recruiting Dnmt1, Dnmt3a, and Dnmt3b [21]. Through a cis-regulatory module, lncMAAT increases the expression of neighboring gene Mbnl1, thereby preventing muscular atrophy [22]. In addition, trans-regulation is another means by which lncRNAs can affect genes distant from their transcriptional sites [23]. Enrichment analysis of trans-target genes in this study also enriched several GO terms and KEGG pathways related to muscle development, including striated muscle tissue development, muscle cell differentiation, MAPK signaling pathway, and calcium signaling pathway, which indicate that lncRNAs are also involved in goat myogenesis by regulating trans-target genes. Previous studies did demonstrate that lncRNAs can function in trans to influence muscle development. For instance, the interaction between lncRNA-FKBP1C and MYH1B enhances the stability of MYH1B protein, thereby regulating myoblast proliferation and differentiation [24]. Moreover, enrichment analysis of antisense lncRNAs was also performed in this study. Interestingly, we found some antisense target genes involved in muscle cell development, such as TGFB2 (the antisense target gene of MSTRG.4012.1) [25] and ATP8B1 (the antisense target gene of MSTRG.9246.1) [26]. Mechanically, antisense lncRNAs may form sense-antisense pairs by pairing with a protein-coding gene on the opposite strand to regulate mRNA stability and translation, thereby influencing the proliferation and differentiation of myoblasts [27,28]. There is evidence that Sirt1 antisense lncRNA preferentially interacts with Sirt1 mRNA, forming an RNA duplex to promote Sirt1 translation by competing with miR-34a, inhibiting muscle formation [29]. Antisense lncRNA MyHC IIA/X-AS was identified in the intergenic region of porcine MyHC IIa and IIx, which functions as a ceRNA to regulate MyHC IIx expression and fast myofiber phenotype [30]. On the whole, these results may provide abundant annotation and reference for the future functional exploration of lncRNA (cis-acting, trans-acting, and antisense mechanisms) in myogenesis and muscle differentiation.

Furthermore, the myogenic regulatory factor (MRF) family, including MyoD, MyoG, Myf5, and Myf6, is a critical muscle-specific regulator that controls skeletal myogenesis [31,32,33]. These four MRF family genes play pivotal roles in the early development of skeletal muscle, where they are sequentially expressed [34]. Therefore, we pay more attention to the interaction pairs related to MyoD and MyoG, while the DE lncRNA-mRNA interaction networks based on cis-action and trans-action were constructed. MyHC is also concerned because it is a skeletal muscle-specific contractile protein expressed during muscle development [35]. On the other hand, accumulating evidence supports the fact that lncRNAs regulate target genes by competitively adsorbing miRNAs, which is termed the ceRNA hypothesis [16,36,37,38]. For instance, linc-MD1, a muscle-specific lncRNA, competes with miR-133 and miR-135, to upregulate the expression of myocyte enhancer factor 2C (MEF2C) and mastermind-like transcriptional coactivator 1 (MAML1), which activate muscle-specific gene expression and regulate myoblast differentiation in mouse and human [39]. To fully identify how lncRNA-associated ceRNA networks affect muscle differentiation in goats, we predicted and constructed the ceRNA networks associated with DE lncRNAs. More importantly, a ceRNA network centered on MyoD and MyoG (containing 25 lncRNAs and 53 miRNAs) and three other ceRNA networks centered on high connectivity lncRNAs were constructed. Based on all the above network analyses, especially the networks we visualized, a large number of MyoD-, MyoG-, and MyHC-related pairs were identified. The lncRNAs involved may have unique functions in myoblast differentiation and skeletal muscle development, which may be the future direction of molecular breeders.

## 4. Materials and Methods

### 4.1. Cell Culture and Sample Preparation

The primary MuSCs were isolated and cultured from *longissimus dorsi* muscle derived from a fetal goat, as described previously [15,40]. The MuSCs were seeded in 6-well plates (at a density of ~2 × 10^4^ cells/well) and cultured in the GM with high-glucose Dulbecco’s modified Eagle medium (DMEM, Gibco, Carlsbad, CA, USA) containing 10% fetal bovine serum (FBS, Gibco, Carlsbad, CA, USA) and 2% antibiotics (including 200 U/mL penicillin and 200 μg/mL streptomycin) (Gibco, Carlsbad, CA, USA). GM was replaced with the DM containing 2% horse serum (HS, Gibco, Carlsbad, CA, USA) and 2% antibiotics to induce myoblast differentiation when MuSCs reached 80-90% confluence. All cells were cultured in a 5% CO_2_ atmosphere at 37 °C. The medium was replaced with fresh medium every 48 h. The MuSCs proliferated were classified as GM samples, while the MuSCs differentiated for 1 day and 5 days were classified as DM1 and DM5 samples, respectively (biological triplicates were included for all of the conditions). All samples were immediately kept at –80 ℃ before RNA extraction.

### 4.2. RNA Extraction, Library Construction, and Sequencing

Total RNA was extracted using Trizol reagent kit (Invitrogen, Carlsbad, CA, USA) according to the manufacturer’s protocol. RNA quality was assessed using the Agilent 2100 Bioanalyzer (Agilent Technologies, Palo Alto, CA, USA) and analyzed by RNase-free agarose gel electrophoresis. The enriched RNAs were fragmented into short fragments by using fragmentation buffer and reverse transcribed into cDNA with random primers. Next, cDNA fragments were purified using the QiaQuick PCR extraction kit (Qiagen, Venlo, The Netherlands), end repaired, bases added, and ligated to the Illumina sequencing adapter. Then, Uracil-N-Glycosylase (UNG) was used to digest the second-strand cDNA. Following digestion, the digested products were size-sorted by agarose gel electrophoresis and sequenced by Illumina HiSeqTM 4000 by Gene Denovo Biotechnology Co. (Guangzhou, China).

### 4.3. Transcriptome Data Analysis

The clean data were obtained by removing reads containing adapters, reads containing over 10% of ploy-N, and low-quality reads containing more than 50% of bases with Q-value ≤ 20 from the raw data. Bowtie2 (version 2.2.8) [41] was used for mapping reads to ribosome RNA (rRNA) database, and the rRNA mapped reads were then removed. All the downstream analyses were based on the high-quality clean data generated in this step. Clean reads for each sample were mapped to the goat reference genome assembly ARS1 (GCA_001704415.1) [42] using HISAT2 (v2.1.0) [43]. The mapped reads from each sample were assembled using StringTie (v1.3.4) [44].

### 4.4. Identification of LncRNAs

To identify new transcripts, all reconstructed transcripts were aligned against the reference genome and classified into 12 categories using Cuffcompare (v2.2.1) [45]. The transcripts with a classcode “u, i, j, x, c, e, o” were defined as novel transcripts. Next, the transcripts with exon number ≥ 2 and transcript length > 200 bp were remained. We then utilized the CNCI (v2) [46] and CPC (v0.9-r2) [47] to predict transcripts with coding potential. Transcripts with a CPC score < −1 and a CNCI score < 0 were eliminated, as well as any transcripts similar to proteins in the Swiss-Prot and Pfam databases (release 33.1). The final possible lncRNA dataset was derived from the transcripts that were identified as non-coding by the intersection of these two methods.

### 4.5. Differential Expression Analysis

Abundances of the transcripts were quantified using StringTie (v1.3.4). The expression was normalized by FPKM using RSEM [48]. DESeq2 [49] was used to analyze the differential expression between the two groups. The transcripts with the parameter of FDR < 0.05 and |log2FC| > 1 were assigned as differentially expressed.

### 4.6. Target Gene Prediction and Enrichment Analysis

Three methods were used to predict target genes for lncRNAs. Cis-target genes of lncRNAs were identified for each lncRNA locus by identifying its 10 kb upstream and downstream protein-coding genes. The trans-regulation of lncRNAs was analyzed by correlation analysis or co-expression analysis of lncRNAs and protein-coding genes, and a Pearson correlation coefficient above 0.95 was considered significant. In addition, RNAplex (v0.2) [50] was used to predict the short interaction between antisense lncRNA and mRNA. Finally, the GO and KEGG enrichment analysis was conducted using the OmicShare tools, an online platform (www.omicshare.com/tools, accessed on 2 August 2022) for data analysis, with the corrected *p* < 0.05 considered significantly enriched.

### 4.7. Construction of DE LncRNA-mRNA and CeRNA Networks during Myogenic Differentiation

The target genes of DE lncRNAs from each comparison were further screened to investigate the interaction between lncRNAs and their target mRNAs. Based on the interaction relationships, myogenesis-related genes and potential lncRNAs were filtered and established into visualized lncRNA-mRNA interaction networks using Cytoscape (v3.8.2) [51]. There is evidence that lncRNAs play a role in regulating gene expression by acting as sponges for miRNAs [17,52]. The target miRNAs, which could be bound to the lncRNAs, were predicted using the mireap (v0.2) [53], miRanda (v3.3a) [54], and TargetScan (v7.0) [55] with a set of default parameters. The target mRNAs of these miRNAs were further predicted. LncRNA-miRNA-RNA regulatory relationships were determined according to the binding relationships between lncRNAs and target miRNAs, as well as binding relationships between miRNAs and target mRNAs. Finally, the regulatory networks were visualized using Cytoscape (v3.8.2).

### 4.8. Quantitative Real-Time PCR

The expression of MyoD, Myogenin (MyoG), and Myosin heavy chain (MyHC) in MuSCs at GM, DM1, and DM5 was measured by quantitative real-time PCR. Additionally, eight lncRNAs were randomly chosen to test the accuracy of RNA-seq using qRT-PCR. Glyceraldehyde-3-Phosphate Dehydrogenase (GAPDH) gene was served as an internal control [56,57]. The primers were designed using Primer-BLAST (http://www.ncbi.nlm.nih.gov/tools/primer-blast/, accessed on 5 May 2021) and are listed in Supplementary Table S15. Total RNA was converted to cDNA using PrimeScript™ RT Reagent Kit (Takara, Tokyo, Japan). The qRT-PCR was performed according to the instructions provided with the SYBR Premix Ex Taq™ II kit (TAKARA, Dalian, China). The PCR system (10 µL) consisted of SYBR Premix Ex Taq 5 µL, 0.4 µL of forward and reverse primers (10 µM), 0.8 µL cDNA, and 3.8 µL ddH_2_O. The relative expression levels of lncRNAs were calculated by the 2-ΔΔCt method [58] and normalized to the control.

### 4.9. Statistical Analysis

Statistical analysis was performed using IBM SPSS Statistics 27.0.0 (SPSS, Chicago, IL, USA). A Student’s *t*-test was used to conduct a comparative analysis of two groups. The figures were prepared using GraphPad Prism 8.0 (GraphPad, San Diego, CA, USA). Differences were regarded as significant at *p* < 0.05 and highly significant at *p* < 0.01.

## 5. Conclusions

In this study, we characterized the lncRNA profile during MuSC proliferation and differentiation by RNA-seq and identified potential lncRNAs that may be involved in the development of skeletal muscle in goats by functional analysis and RNA interaction network analysis. This work provides a catalog of lncRNAs expressed in goat myoblasts that will facilitate understanding their regulatory functions during myogenic differentiation, as well as a list of candidate lncRNAs for further study of goat skeletal myogenesis.

## Data Availability

The datasets presented in this study can be found in online repositories. The names of the repository/repositories and accession number(s) can be found below: https://www.ncbi.nlm.nih.gov/bioproject/ (accessed on 10 November 2021), PRJNA779184.

## Supplementary Materials

The following supporting information can be downloaded at: https://www.mdpi.com/article/10.3390/ijms24076370/s1.

## Abbreviations

ceRNA	Competing endogenous RNA
CNCI	Coding-Non-Coding-Index
CPC	Coding Potential Calculator
DE	Differentially expressed
DM	Differentiation medium
FC	Fold change
FDR	False discovery rate
FPKM	Fragments per kilobase of transcript per million fragments mapped
GAPDH	Glyceraldehyde-3-phosphate dehydrogenase
GM	Growth medium
GO	Gene Ontology
KEGG	Kyoto Encyclopedia of Genes and Genomes
LncRNA	Long non-coding RNA
MuSCs	Skeletal muscle satellite cells
MyHC	Myosin heavy chain
MyoD	Myogenic differentiation 1
MyoG	Myogenin
qRT-PCR	Quantitative real-time PCR
