# Supplementary figures and images for: Transcriptome Analysis Reveals the Profile of Long Non-Coding RNAs during Myogenic Differentiation in Goats
Source: Int J Mol Sci. 2023 Mar 28;24(7):6370. doi: 10.3390/ijms24076370 (PMC10094361; doi:10.3390/ijms24076370)

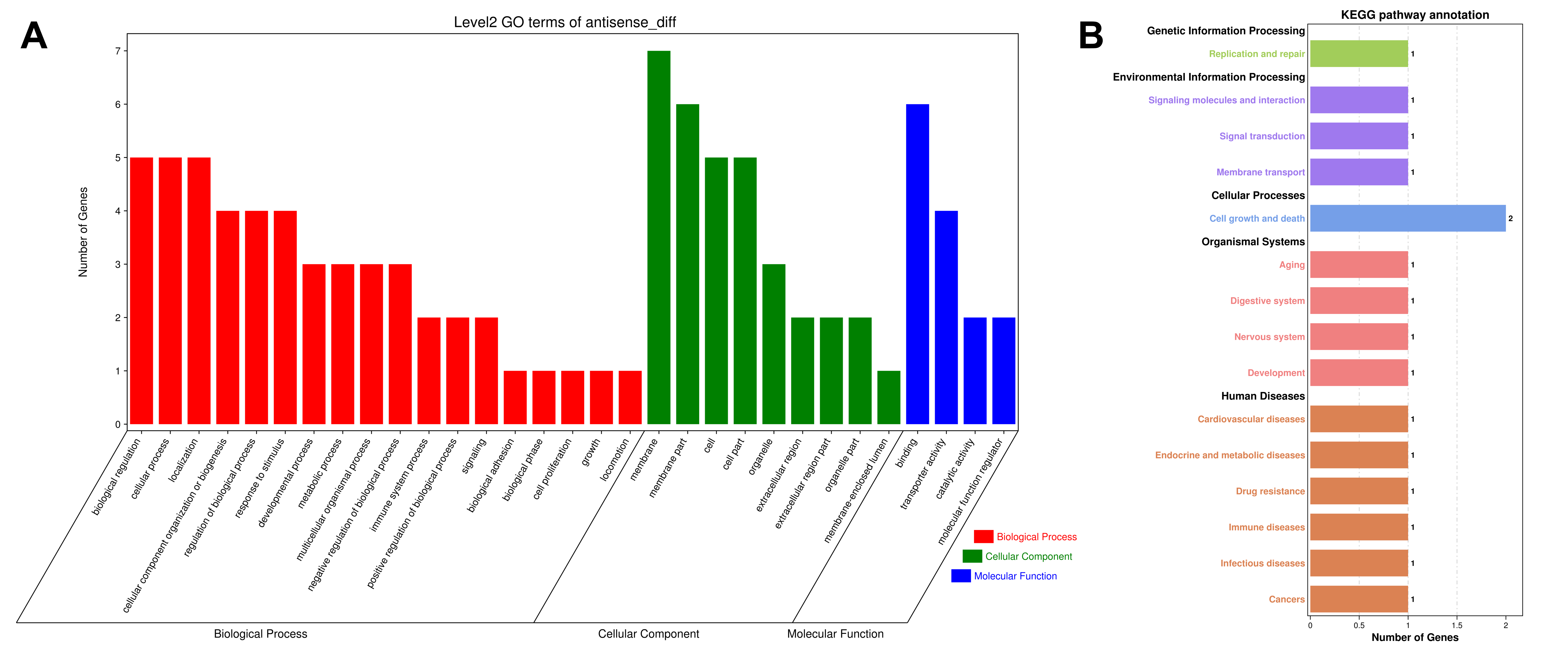

Supplement: Supplementary file 1 [file ijms-24-06370-s001.zip › Figure S1. Enrichment analysis of antisense-target genes of DE lncRNAs.png]
